# Comparison of software-assisted and freehand methods of rotational assessment for diaphyseal femur fractures

**DOI:** 10.1007/s00590-024-04121-3

**Published:** 2024-12-21

**Authors:** Christian Blough, Kevin Huang, John Garlich, Milton Little, Charles Moon, Geoffrey Marecek

**Affiliations:** Department of Orthopaedic Surgery, Cedars-Sinai Medical Center, 444 S. San Vincente Blvd, Suite 603, Los Angeles, CA 90048 USA

**Keywords:** Femur fracture, Rotational assessment, Software augmentation

## Abstract

**Objective:**

Accurate rotational reduction following femoral shaft fracture fixation is absent in up to 28% of cases yet is critical for lower extremity biomechanics. The objective of this cadaveric study was to compare the results of freehand methods of rotational reduction with software-assisted rotational reduction.

**Methods:**

Four fellowship-trained orthopedic trauma surgeons attempted rotational correction in a cadaveric model with fluoroscopic assistance using (1) their method of choice (MoC) and (2) software assistance (SA). After correction, deviation from baseline rotation was calculated.

**Results:**

The mean difference between the two methods (MoC–SA) was 1.1 which was not significant when comparing all raters and between raters individually. SA had significantly less variability compared to MoC. The rate of clinically relevant rotational deformity (> 15°) was 28% using MoC and 11% using SA.

**Conclusion:**

Rotational assessment of diaphyseal femur fractures in this cadaveric model was not significantly different when compared between method of choice and software augmentation.

## Introduction

The incidence of diaphyseal femur fractures worldwide is high, estimated to be 10–21 per 100,000 persons per year [1–3]. The standard of care when managing diaphyseal femur fractures in adults is intramedullary nailing. Although intramedullary nailing is generally a very successful procedure, inadequate reduction prior to, and during fixation, can lead to postoperative deformity associated with pain and decreased function. Of the common deformities, rotation is the most difficult to assess clinically and radiographically [4]. This is especially true in certain fracture patterns where cortical alignment is unable to be assessed. Proper rotational alignment is critical to restore lower extremity biomechanics, but adequate alignment (i.e., less than 15 degrees of rotational deformity) is not achieved in up to 28% of cases for femoral shaft fractures [5]. If rotational alignment is not properly achieved, the risk for short-term complications like persistent pain and inability to perform desired activities, as well as long-term complications, like knee and ankle osteoarthritis, is increased [6–8]. While the importance of restoring rotational alignment is acknowledged, there is no clear consensus on the best method for achieving this [9, 10]. Further, only 63% of surgeon are able to correctly identify significant malrotation, described as greater than 20 degrees of rotation relative to the contralateral limb, on intraoperative radiographs of femoral shaft fractures [11].

A novel fluoroscopy-based software program (Surgeon Checklist Trauma, RadLink, El Segundo, California) can be used to assess rotation intraoperatively. The software can automatically detect bony landmarks and then overlay images with the bony landmarks outlined to assist in rotational reduction of fractures. If this software can improve surgeons’ ability to assess rotational alignment intraoperatively compared to current techniques, it could be a valuable tool in the management of diaphyseal femur fractures.

The purpose of this study is to determine if software assistance can improve rotational reduction in femoral shaft fractures compared to currently used techniques. We hypothesized that software assistance would improve rotational reduction in diaphyseal femur fractures.

## Materials and methods

Five matched pairs of hip-to-toe cadaveric specimens without history of lower extremity injury or surgery were obtained (Science Care, Phoenix, AZ). Each cadaveric lower extremity underwent computed tomography (CT) scanogram. The femoral torsion was then calculated using the method described by Hernandez et al. [12].

One side was randomly chosen to be the fractured extremity using a random number generator. Schanz pins were placed in the proximal and distal femoral metaphyses at random orientations in the axial plane. The angle difference from horizontal (i.e., the plane of the surgical table) of each Schanz pin was recorded with an electronic level after placement. The difference in angles was calculated and recorded as the baseline. Next, a transverse osteotomy was performed in the midshaft of the chosen femur to mimic a femoral shaft fracture. A guidewire was then placed in a retrograde fashion into the medullary canal of fractured side to simulate reduction with a medullary nail intraoperatively.

Four fellowship-educated orthopedic traumatologists with a mean of 11-year experience (range 2–22 years) were prompted to rotationally reduce the fracture in each specimen with two techniques. The first technique would be the method of their choice (MoC). The second technique would be using software assistance (SA). They were provided with a C-arm, an assistant for manipulating the C-arm, a tower containing a screen and the computer running the software program, as well as a representative to assist with the software. Surgeons were not permitted to visualize the fracture site fluoroscopically. This was done to ensure that we were evaluating a specific method of rotational reduction, and not a summation of methods.

**Fig. 1:**
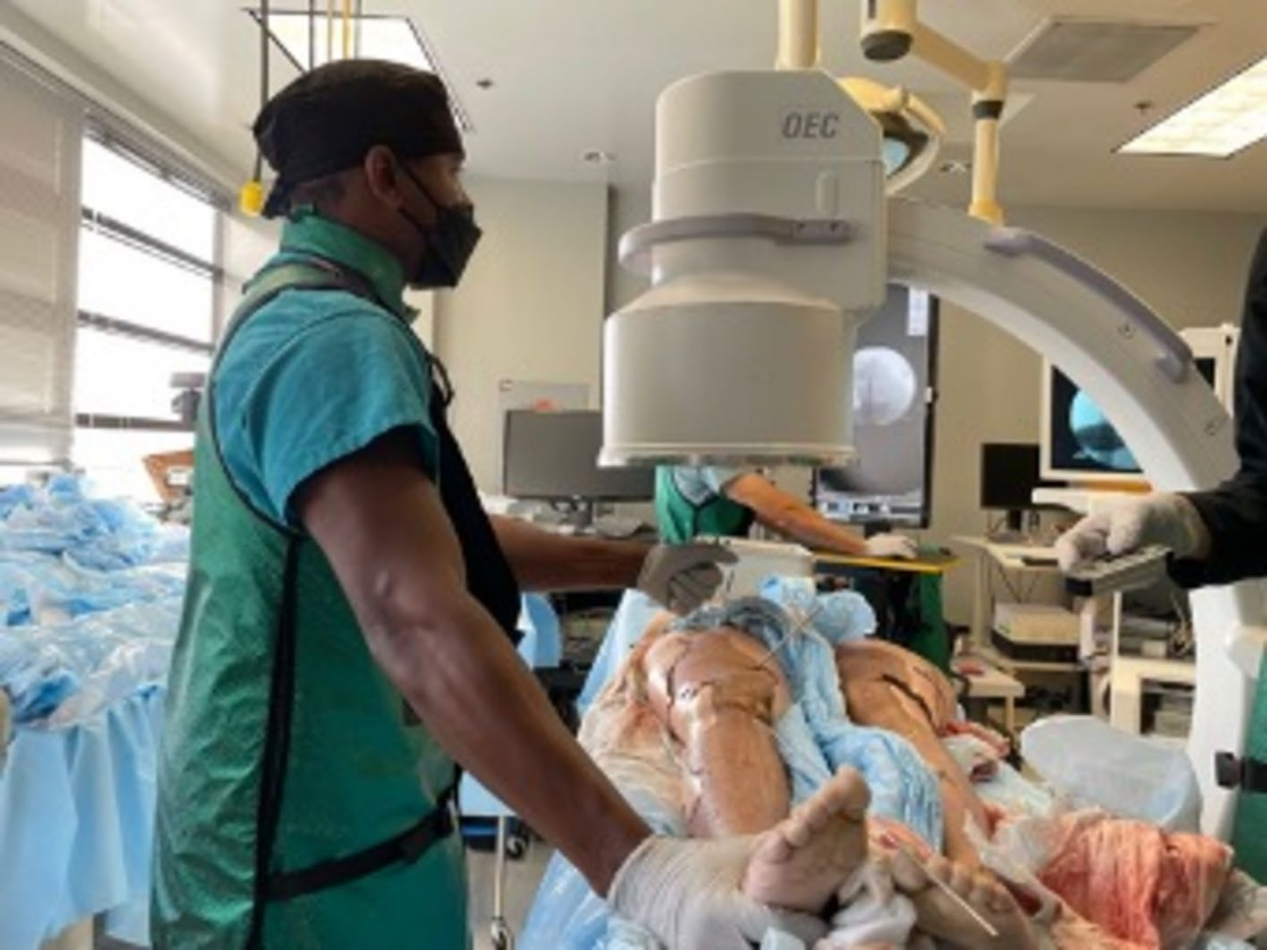
Experimental Setup

To begin each trial, the matched hip-to-toe cadaveric specimens were placed supine onto an operating room table. The rotation about the fracture on the fractured side was then randomly manipulated before the surgeon was allowed to enter the room, thus blinding them to the “correct” orientation of the Schanz pins. The surgeon was then asked to reduce the fracture using their MoC (Fig. 1). When they were satisfied with their reduction, the angle between each Schanz pin and horizontal was again measured and recorded. The difference between these two angular measurements was then calculated and compared to the value prior to osteotomy to determine the rotational malreduction relative to the ipsilateral limb. This was done for each of the five cadaver pairs. Between each trial the rotation of the Schanz pins was randomly manipulated.

All four of the trauma surgeons chose to use the lesser trochanter profile method of assessing femoral rotation as their MoC. The lesser trochanter profile is an intraoperative method of assessing femoral torsion which was first described by Dunlap et al.[13]. One first obtains a lateral radiograph of the knee on the uninjured limb. The C-arm is then rotated 90°, and a radiograph of the hip is obtained. The profile of the lesser trochanter on this hip radiograph is used as a template to for the contralateral limb. After obtaining a lateral knee radiograph of the injured limb, the C-arm is again rotated 90° and the radiograph of the injured limb’s hip is obtained. The profile of the lesser trochanter on this view is then made to match the contralateral profile while maintaining the rotation of the distal femoral segment. This method has been widely accepted despite the fact that the rate of malrotation remains high [5].

**Fig. 2:**
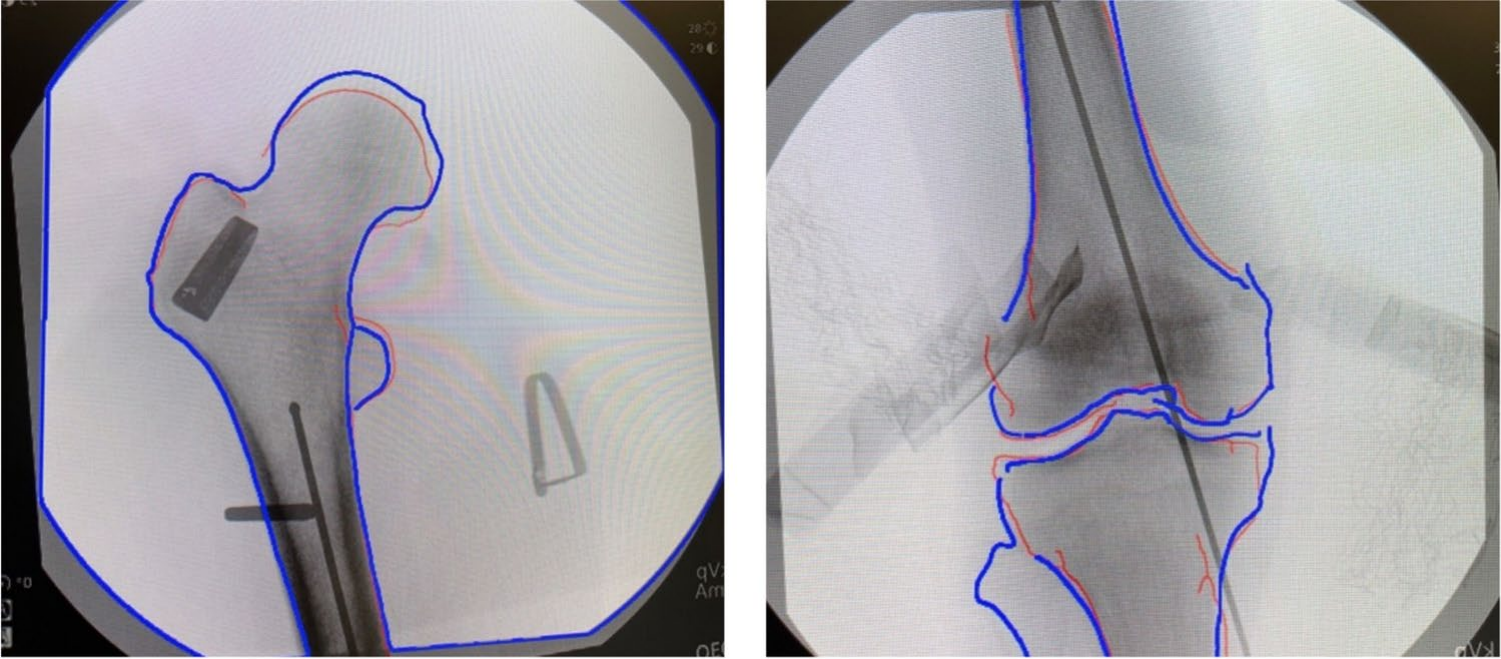
Image Overlay using Software Assistance

Next, this protocol was repeated with each surgeon using SA to assist in their rotational reduction. Each surgeon chose anteroposterior (AP) fluoroscopic views of the proximal and distal femur to assess rotation. A baseline shot of the intact proximal and distal femur was taken and recorded. The cortices of boney landmarks about the knee and hip from these fluoroscopic images of the intact limb were then outlined with the assistance of the software. This process is completed first by the software, and subsequent corrections can be made by hand. This outline was then mirrored to match the injured limb and displayed. The surgeon then attempted to match the AP view of the knee on the injured limb with the displayed, outlined image. Once satisfied with the fluoroscopic image of the injured limb, the image was uploaded to the software, and the cortices of boney landmarks surrounding the knee on the injured limb were outlined in the same fashion as previously described and overlaid with the intact femur’s outline (Fig. 2). If satisfied with the overlaid outlines of the distal femur/knee, they then corrected the rotational deformity of the injured side by matching the displayed, outlined view, of the intact proximal femur with the fluoroscopic view of the injured limb. This fluoroscopic image was then uploaded to the software, the boney landmarks were outlined, and the outlined images of the proximal femurs were overlaid (Fig. 2). The surgeon was then asked if they were satisfied with their rotational correction. If they were, the angle between each Schanz pin and horizontal was again measured in a similar fashion. This was again done for each of the five cadaver pairs. Between each trial the rotation of the Schanz pins was randomly manipulated.

The rotational deformity of the injured femur following rotational reduction was then compared to the ipsilateral, native femur prior to the simulate fracture, and the contralateral, matched femur, using the difference in baseline torsion known from the baseline CT scanogram. Comparison to the contralateral femur was done to simulate clinical practice, in which the uninjured contralateral femur is available for comparison and is the surrogate for correction of the injured limb. We report both comparisons as we feel that it is important to evaluate rotational reduction methods relative to the native, pre-injured femur, and the contralateral femur which is often used as a surrogate for the native, pre-injure femur’s torsion.

Surgeons performed each assessment twice separated by several weeks. This totaled 40 reductions for the MoC and SA group each. The average measurement across the two study sessions was calculated for each specimen (femur), surgeon, and method combination. This average rating was used for comparisons between methods, surgeons, and specimens. The rotation variables were identified as not normally distributed based on the Shapiro–Wilk test. For this reason, nonparametric tests were used to evaluate the difference in malrotation by method (Mann–Whitney U test), by surgeon (Kruskal–Wallis test), and by specimen (Kruskal–Wallis test).

To assess clinically relevant differences categorical rotational deformity, we performed Fisher’s exact tests to compare across the two methods. Furthermore, we compared the variability of the two methods using an F test.

## Results

The demographics and baseline characteristics of each femur can be found in Table 1. The average age of death for the donors was 75.2 years old (range 55–89). Four (80%) of the donors were female, and one was male. The average femoral torsion was 10.2° (± 13) of internal rotation (antetorsion). The average difference in torsion between matched femurs was 9.6° (range 4–16°).

**Table 1:** Demographics and Baseline Torsion

Specimen number	Sex	Age at death (years)	Left antetorsion (degrees)	Right antetorsion (degrees)	Antetorsion difference (Left—Right)
**1** & 2	Female	89	5	**21**	− 16
**3** & 4	Female	55	− 1	**6**	− 7
5 & **6**	Female	83	**29**	24	5
**7** & 8	Male	74	23	**7**	16
**9** & 10	Female	75	− 4	**-8**	4

Bolded numbers are the “injured” laterality

When comparing relative the ipsilateral limb, evaluating restoration of native anatomy, the mean rotational deformity following reduction with MoC ranged from 2.2° to 9.4° by surgeon and from 1.6° to 14.4° by specimen. For SA, the mean rotational deformity following reduction ranged from 5.9° to 7.2° by surgeon and from 5.3° to 8.0° by specimen (Table 2).

**Table 2:** Average Rotational Deformity

		Surgeon				Specimen				
		1	2	3	4	1	3	6	7	9
Versus ipsilateral	MoC	2.2°	8.5°	5.6°	9.4°	14.4°	9.5°	4.3°	1.6°	2.6°
	SA	5.6°	7.2°	6.7°	7.1°	8.0°	5.3°	7.6°	5.3°	7.5°
Versus contralateral	MoC	8.8°	7.3°	4.9°	8.1°	7.8°	5.5°	3.7°	14.9°	4.3°
	SA	8.4°	8.0°	10.4°	8.8°	16.3°	6.6°	4.3°	13.3°	4.0°

Average malrotation displayed as absolute value

When comparing relative to the contralateral limb, as is often done in clinical practice intraoperatively, the mean rotational deformity following reduction with MoC ranged from 4.9° to 8.8° by surgeon and from 3.7° to 14.9° by specimen. For SA, the mean rotational deformity following reduction ranged from 8.0° to 10.4° by surgeon and from 4.0° to 16.3° by specimen (Table 2).

**Fig. 3:**
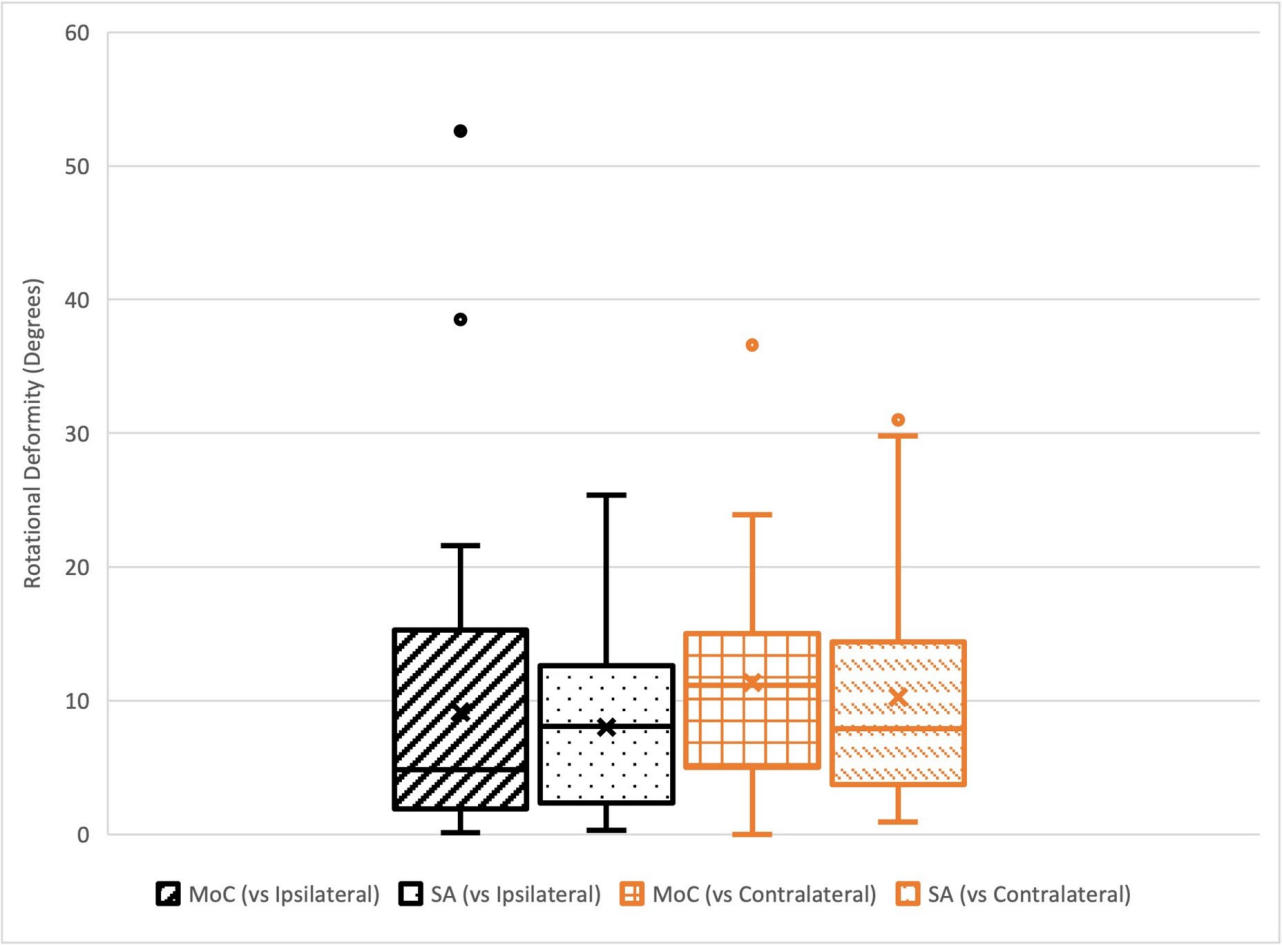
Rotational Deformity by Method Relative to Contralateral Extremity (MoC—Method of Choice, SA—Software Assistance)

The mean rotational deformity (absolute value) compared to the ipsilateral femur after correction with MoC was 9.1°± 10.7°(range 0.1–52.6°) and with SA was 8.0°±5.8°(range 0.3–25.4°); no significant difference was noted between methods (*p* value 0.78). Relative to the contralateral femur, the mean rotational deformity (absolute value) after correction with MoC was 11.3°± 7.7°(range 0.0–36.6°) and with SA was 10.3°±8.0°(range 0.9–31.0°); again, no significant difference was noted between methods (*p* value 0.43). There was also no significant difference noted when comparing methods by surgeon or specimen (*p* values > 0.05). The mean difference in rotational deformity (MoC–SA) relative to the ipsilateral femur was 1.1°and relative to the contralateral femur was 1.0° (Fig. 3). Both of these differences were less than our calculated effect size.

Categorical comparison of rotational deformity by 5° intervals can be found in Table 3. Fisher exact test results did reveal a significant difference between methods (MoC vs SA) relative to ipsilateral femur overall (*p* value 0.01) but not when comparing rotational deformity of ≤ 15° to rotational deformity of > 15°(*p* value 0.08). Fisher exact test results did not reveal a significant difference between methods relative to the contralateral femur.

**Table 3:** Categorical Comparison of Rotational Deformity

Malrotation (degrees)	Vs. Ipsilateral		Vs. Contralateral	
	Method of Choice	Software Assistance	Method of Choice	Software Assistance
	N (%)	N (%)	N (%)	N (%)
< 5	20 (50)	15 (38)	10 (25)	13 (33)
5.0–10	7 (18)	8 (20)	7 (18)	10 (25)
10.1–15	2 (5)	13 (33)	13 (33)	8 (20)
15.1–20	7 (18)	3 (8)	4 (10)	3 (8)
> 20	4 (10)	1 (3)	6 (15)	6 (15)

*Absolute value of rotational deformity used for categorical comparison*

In assessing the variability of rotational deformity between methods (MoC vs SA), relative to the ipsilateral femur, the F test value was 3.4 (*p* value 0.0002) indicating that the SA method has less variability. Relative to the contralateral femur, the F test value was 0.93 (*p* value 0.83), indicating no difference in variability of measurements between method.

## Discussion

Software assistance produced comparable results compared to the lesser trochanteric profile method for assessing torsional reduction of diaphyseal femur fractures in this cadaveric study. These results should be reflective of the general population as our specimens had baseline torsion within the established range of 7–24° [14].

Both the lesser trochanter profile method and the SA method rely on an uninjured contralateral limb to assess rotation reduction. They also rely on the assumption that anatomic features and femoral torsion between limbs is consistent. Croom et al. investigated the individual bilateral difference in femoral torsion with CT scans, finding that approximately 18% of individual had > 10° of individual bilateral difference and ~ 4% of individuals having > 15° of individual bilateral difference. They also noted that > 20° of antetorsion or retrotorsion on one limb increased the likelihood of the individual bilateral difference being large [15]. This indicates that care must be taken when only using the contralateral limb for rotational correction given the possibility of a large individual bilateral difference. Two of the specimen pairs (1–2 and 7–8) in this study had a 16° difference in torsion between literalities. Given that most rotational reduction techniques use the contralateral limb as a baseline to compare to, this large of a difference in torsion leaves little room for error for the surgeon before a clinically significant rotational difference is present between limbs.

The large difference in native torsion in two of our specimens did not appear to have an effect on the rotation correction between methods. When comparing MoC vs. SA by cadaveric specimen, there was no significant difference found.

A recent cadaveric study comparing the lesser trochanter profile between limbs found that if properly performed, the profile is similar in most patients, except for elderly females [16]. In assessing rotation with the lesser trochanter profile, all surgeon’s average malrotation was less than 15°, the degree of malrotation which is thought to be clinically relevant [5]. As shown in Table 6, there were 11 reductions that exceeded this 15° threshold relative to the ipsilateral femur, and 10 relative to the contralateral femur. This represents a 28% and 25% rate of clinically significant rotational deformity. It is possible that a high prevalence of elderly female specimens in our study may have affected the success of the surgeons’ MoC. Surgeons were not informed of patient demographics during the study.

RadLink’s “Surgeon Trauma Checklist” utilizes tracing of cortical bone landmarks on multiple fluoroscopic images, which can then be overlaid to assess for symmetry when obtaining fracture reduction. The software theoretically allows a more comprehensive rotational assessment given that additional bone landmarks are traced besides the lesser trochanter. When using SA for assessing reduction, all four surgeons’ average malrotation was again less than 15°. There were four reductions that exceeded the 15° threshold relative to the ipsilateral femur, and nine reductions relative to the contralateral femur. This represents an 11% and 23% rate of clinically relevant rotational deformity, slightly less than the rate using MoC, especially relative to the ipsilateral limb. All surgeons chose AP fluoroscopic images to assess rotation using software assistance to utilize the lesser trochanter profile and proximal tib–fib overlap and patellar position as guides to rotation. We cannot determine if the technique would be more or less accurate using lateral fluoroscopic images or a combination of lateral and AP images, though it may warrant further investigation. A lateral image of the knee is used during standard rotational assessment because it is clearly defined and reproducible (unlike an AP image of the knee); use of software assistance may obviate the knee for lateral fluoroscopic imaging. Lateral fluoroscopic imaging has been identified as a potential source of error during rotational assessment due to parallax [17].

Positioning of the patient during intramedullary nailing of femoral shaft fractures is commonly completed supine or lateral. Supine positioning, often on a fracture table, allows constant traction with the limb held motionless. It is also advantageous for polytraumatized patients with chest or spine injuries. Lateral decubitus positioning allows for the entry point to be accessed easier and the entire extremity to be draped free. Additionally, lateral decubitus positioning has been purported to make for more difficult rotational control for inexperienced surgeons. Sholla et al. investigated diaphyseal femoral fracture nailing in both supine and lateral decubitus nailing and found no difference in rotational reduction [18]. The supine position was used in this study as that is the preferred position at our institution.

All the surgeons in this study were fellowship-educated, practicing traumatologists. Ayalon et al. previously investigated if fellowship training effected rotational reduction in diaphyseal femur fractures [19]. They did not find a significant difference in postoperative femoral torsion when comparing surgeons but did argue that a prospective trial where traumatologists are not completing more complex cases is needed. We share this viewpoint and believe that software assistance may be of increased benefit to surgeons who are not treating diaphyseal femur fractures as often. This is because cases with significant malrotation may require revision surgery to correct rotation, future surgery to address the sequelae of rotational deformity, and overall increased healthcare expenditure and potential litigation.

The surgeons in our study were not permitted to view the osteotomy site to simulate a situation in which extensive comminution precluded direct reduction or cortical reads. This theoretically isolates the utility of each technique on its own. In practice, surgeons typically combine techniques and thus the degree of error in this study may be an overstatement of true clinical practice. However, a recent study used a nearly identical methodology to compare multiple established methods of assessing rotation, establishing this methodology as viable [20].

There are several limitations to this study. The first is that it was completed on cadaveric specimens in which fractures were simulated. Second, due to laboratory, equipment, and surgeon availability, multiple days were needed to complete the study. This resulted in the specimens undergoing multiple freeze–thaw cycles which can affect the soft tissues, thereby potentially altering the ease of obtaining and maintain reduction. Additionally, because of the degradation from multiple freeze–thaw cycles and the time-consuming nature of completing the study, we were limited to having four orthopedic traumatologists complete the study. Although a study with a larger number of specimens and observers would be ideal, two recent studies used a similar number of specimens and equal or fewer observers [20, 21]. Next, surgeons were provided a minimal introduction to the software package prior to use and some had not used the software during cases previously; a learning curve may be present that would allow improved results over time with software assistance. Finally, all surgeons in this study are traumatologists who routinely treat femoral shaft fractures. Other orthopedic surgeons, who treat this injury less commonly, may produce different results in a similar study.

Given the mean difference relative to the ipsilateral limb between methods found in our study (1.1°), we calculated that a study with 80% power to detect an alpha value of 0.05 would need 883 reductions using each method to detect this effect size. This indicates our study in underpowered, but a study of this size is likely not viable using a cadaveric model. We additionally calculated what the number of reductions would be needed using each method to detect a clinically significant difference between methods of 10°. Twelve reductions using each method were necessary to detect this difference; therefore, our study with 20 measurements using each technique was adequately powered by this metric.

## Conclusion

Rotational reduction for diaphyseal femur fractures treated with medullary nails can be challenging, with rotational deformity leading to short- and long-term complications. Software-assisted technology was investigated as a possible method to improve rotational assessment. In this cadaveric study, there was no difference found in the assessment of rotational reduction of diaphyseal femur fractures with surgeons’ method of choice compared to software assistance. Given the high rate of femoral shaft fractures worldwide, further research in vivo is needed to determine the usefulness and applicability of software assistance for rotational assessment.
